# Acute Type A Aortic Dissection Diagnosed by POCUS in a 29-year-old Man

**DOI:** 10.24908/pocus.v8i2.16533

**Published:** 2023-11-27

**Authors:** Vladimir Cárdenas López, Pablo Blanco

**Affiliations:** 1 Intensive Care Unit, Hospital "Dr. Miguel Belascuain" Concepción Argentina; 2 High-dependency Unit, Hospital "Dr. Emilio Ferreyra" Necochea Argentina

**Keywords:** Aortic Dissections, Point-of-Care, Ultrasonography, Marfan Syndrome

## Abstract

Aortic dissection (AD) is a medical emergency with a poor prognosis if not recognized early and treated promptly. In this setting, clinical data may be equivocal, while electrocardiogram, laboratory tests, and chest radiography often show nonspecific findings. In contrast, cardiac point of care ultrasound (POCUS) has proven useful in the diagnosis and detection of complications of AD. We present the case of a 29-year-old man with marfanoid habitus presenting with chest pain and acute heart failure, in whom cardiac POCUS aided in the rapid diagnosis of type A AD and pulmonary edema. POCUS contributed to optimizing the medical treatment and allowed for early activation of the surgical team.

## Introduction

Aortic dissection (AD) is the most common type of acute thoracic aortic syndromes, compared to intramural hematoma and penetrating atherosclerotic ulcer 1. It has an incidence of 5- 30 cases per million people per year and is more common in men 1. Known risk factors include arterial hypertension, thoracic aortic aneurysm, bicuspid aortic valve, and genetic conditions affecting the tunica media, such as Marfan or Ehlers-Danlos syndrome, aortitis, pregnancy, trauma, and iatrogenia 1. Based on the Stanford classification, dissection including the ascending aorta is known as Type A (A-AD), and dissection not including the ascending aorta is known as type B (B-AD). Two-thirds of dissections are A-AD 2. Early surgery is mandatory in A-AD, whereas B-AD is often treated medically, unless it ruptures or causes malperfusion syndromes 2.

Patients with AD typically present severe chest pain. In addition, depending on the propagation of the dissection, patients may show features of heart failure, myocardial infarction, tamponade, shock, or malperfusion syndromes. These overlapping presentations may confound the attending physician and delay the diagnosis or may even pose a patient´s risk if some treatments are indicated. For instance, there is further urgency of diagnosing aortic dissection as an etiology of stroke as early thrombolytic therapy may be indicated.

To diagnose AD, clinical findings alone may be equivocal, and electrocardiogram, laboratory tests or chest radiography often show nonspecific findings. Cardiac POCUS (transthoracic) can be considered the preferred tool for screening patients with suspected AD given its acceptable diagnostic accuracy (particularly in A-AD), its quickness, non-invasiveness, lack of ionizing radiation, and widespread availability in acute care settings 1. In addition to AD diagnosis, POCUS may aid in the detection of complications, such as aortic regurgitation, acute heart failure, or tamponade. 

## Case presentation 

**Figure 1:**
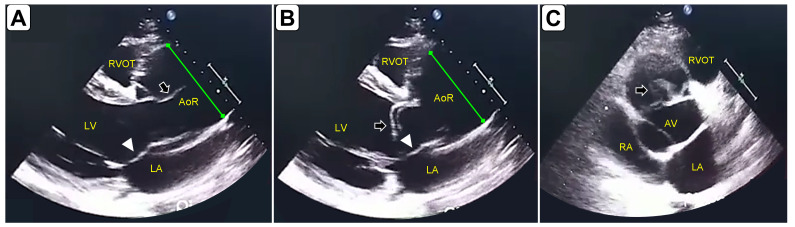
Cardiac point-of-care ultrasound (POCUS) showing signs of type A aortic dissection (A-AD). A. Parasternal long axis (PLAX) view in a systolic frame. B. PLAX view in a diastolic frame. C. Parasternal short-axis view. Arrow indicate the intimal flap, while the continuous green line indicate the aortic root dilation (pseudo-double aortic valve). Arrowhead is pointing to the anterior leaflet of the mitral valve. LV: left ventricle; LA: left atrium; RVOT: right ventricular outflow tract; RA: right atrium;AoR: aortic root; AV: aortic valve.

**Figure 2:**
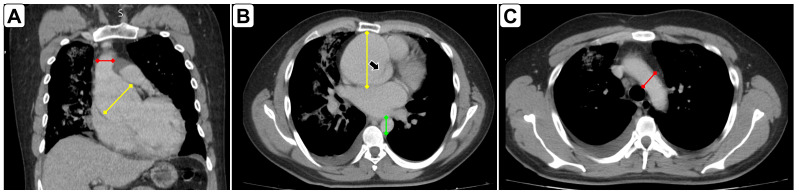
Confirmation of A-AD by chest computed tomography with intravenous contrast medium. A. Coronal plane showing a dilated aortic root/ascending aorta (continuous yellow line) and a normal aortic arch (continuous red line). B. Axial plane showing a dilated ascending aorta (continuous yellow line) with an intimal flap indicated by the arrow, and the normal descending aorta (continuous green line). C. Axial plane at the level of the normal aortic arch (continuous red line).

**Figure 3:**
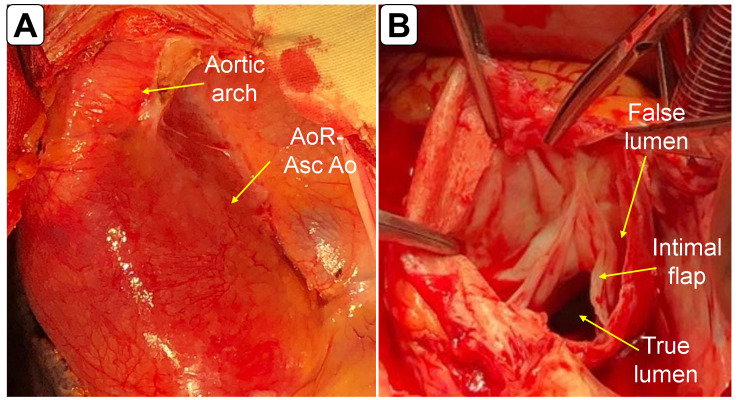
Operative findings of A-AD. A. Aortic root/ascending aorta dilatation and a normal aortic arch are observed. B. The intimal flap and a true and false lumen are demonstrated by opening the aortic root.AoR: aortic root; Asc Ao: ascending aorta.

A 29-year-old man was admitted to the Emergency Department (ED) with chest pain radiating to the right shoulder, progressively worsening over the last five days, accompanied by dyspnea at rest, diaphoresis, and emesis. Vital signs were: heart rate 118 beats per minute; respiratory rate 30 breaths per minute, blood pressure 110/75 in both arms, oxygen saturation 80% on room air; and temperature 36°C. A physical examination revealed a severely distressed patient with excruciating chest pain, orthopnea, and diffuse bilateral crackles on chest auscultation. He had marfanoid habitus. Surface electrocardiography showed sinus rhythm at 118 QRS complexes per minute, with a 1 mm ST level depression in leads DII, DIII, and AVF, and 2 mm from leads V4 to V6. Cardiac POCUS (transthoracic) was performed on arrival. In the parasternal long-axis view, POCUS showed a severely dilated aortic root with a long intimal flap originating from its anterior aspect, protruding into the left ventricle in diastole, and touching the anterior leaflet of the mitral valve (Figure 1A,1B and Video S1). The left ventricle was dilated, and systolic function was severely impaired (Video S1). Severe acute aortic regurgitation was also observed. In the short axis, a pseudo- double aortic valve was mimicked with the pseudo valve as the intimal flap (Figure 1C and Video S2). Diffuse bilateral B-lines confirmed acute pulmonary edema. Chest computed tomography with intravenous contrast medium confirmed an A-AD limited to the aortic root and ascending aorta (Figure 2). The patient was transferred to the operating room, where the preoperative findings were also confirmed (Figure 3). He successfully underwent the Bentall procedure and continued his care in the intensive care unit.

## Discussion

The case presented here is one of several reported cases that highlight the value of cardiac POCUS in diagnosing A-AD without delays in the Emergency Department, leading to improved patient care. The diagnostic accuracy of cardiac POCUS is better for A-AD than B-AD. For A-AD, the sensitivity is 78-100%, whereas for B-AD it is 31–55% 2. In cases where the diagnosis is unequivocal on cardiac POCUS and the patient is unstable, the patient should go directly to the operating room without further imaging techniques 3. However, when cardiac POCUS does not rule out AD and suspicion remains high, advanced imaging techniques such as transesophageal echocardiography (TEE) in unstable patients, chest computed tomography (the most common modality in practice), or magnetic resonance imaging in stable patients should be performed 2, 3, 4, 5, 6. 

Signs of dissection on Cardiac POCUS include aortic dilation and an intimal flap separating the aortic lumen in a true and false lumen. When evaluating for an intimal flap, physicians should be aware of mimickers such as artifacts resembling a double aortic lumen. Reverberating and side-lobe artifacts are often observed in the ascending aorta and aortic root, respectively, leading to false-positive AD diagnosis 7. Regional wall motion abnormalities and pericardial effusion may occur in cases where dissection includes the coronary ostia and pericardium, respectively. Aortic regurgitation of varying degrees can also be observed, as well as B lines, as a sign of pulmonary edema.

The clinical data and phenotypic features of our patient raised the suspicion of A-AD. Dilation and dissection limited to the aortic root/ascending aorta are typical of Marfan syndrome 6, which was easily observed by cardiac POCUS, leading to early offering a definite treatment. In our patient, a chest CT was performed to confirm AD, which seemed unnecessary, and the patient could be transferred to the operating room without the need for advanced imaging techniques 3. 

## Conclusion

The case presented here is a clear example of the paramount importance of cardiac POCUS in the diagnosis of aortic dissection, leading to the early initiation of medical treatment and activation of the surgical team to reach a definite treatment. Without POCUS, the diagnosis could be delayed or even missed, obscuring the patient prognosis. 

## Statement of ethics approval/consent

Complete written informed consent was obtained from the patient´s next of kin for the publication of this case file. 

## Disclosures

This work has not been presented at any conferences and has not been supported by any grants. All authors have no conflicts of interest to disclose.

## Supplementary Material

Video S1. Parasternal long-axis view showing dilation of the aortic root and intimal flap (type A aortic dissection).

Video S2. Parasternal short-axis view showing a pseudo-double aortic valve (type A aortic dissection).

## Supplementary Material

 Video S1 Parasternal long-axis view showing dilation of the aortic root and intimal flap (type A aortic dissection).

## Supplementary Material

 Video S2 Parasternal short-axis view showing a pseudo-double aortic valve (type A aortic dissection).
